# Histologic Evaluation of Three Treatment Methods for Direct Pulp Capping of Cat’s Canine

**Published:** 2007-07-05

**Authors:** Seyed Mohsen Hasheminia, Ghader Feizi, Seyed Mohammad Razavi, Mahboobe Feizianfard

**Affiliations:** 1 *Department of Endodontics, Dental School, Isfahan University of Medical Sciences, Isfahan, Iran*; 2 *Endodontist, Torabinejad Research Centre, Dental School, Isfahan University of Medical Sciences, Isfahan, Iran*; 3 *Department of Oral and Maxillofacial Pathology, Dental School, Isfahan University of Medical Sciences, Isfahan, Iran*

**Keywords:** Calcium Hydroxide, Laser, MTA, Pulp Capping

## Abstract

**INTRODUCTION:** Direct pulp capping (DPC) is coverage of exposed pulp by a biocompatible material after traumatic or carious exposure. The purpose of this procedure is to seal against bacterial leakage, stimulate dentinal barrier formation, and maintain the vitality of pulp. Several factors contribute to the consequence of this treatment such as material and the procedural technique. The aim of this study was to histological evaluation of three treatment methods (Laser+MTA, Laser+Ca(OH)_2_ and MTA alone) in direct pulp capping of cat's canines.

**MATERIALS AND METHODS:** Thirty six canine teeth of 9 cats were selected for this experimental study. After anesthesia, the teeth were exposed under isolated condition. The teeth were randomly divided into three treatment groups. In group I, the pulp exposures were covered by Mineral Trioxide Aggregate (MTA) alone. In group II, the pulps after treating with Er: YAG laser, were covered by MTA. In group III, treating with laser and covering with Ca(OH)2 was performed. All cavities were filled by Amalgam after DPC. After 4 months, the animals were sacrificed and block sections were prepared. Then, the specimens were histologically evaluated according to the scores that designed by a pathologist. The data was analyzed by Mann-Whitney and Chi-square tests with significant level of 95%.

**RESULTS:** Dentinal barrier was formed in all groups. Laser+MTA group showed nearly similar results to other groups in dentinal barrier formation, type and intensity of inflammatory responses and soft tissue changes (*P*>0.05).

**CONCLUSION:** Although Laser+MTA had slightly better effects, but this difference was not statistically significant. Based on this study, it seems that laser treatment has no effect on outcome of DPC.

## INTRODUCTION

Direct pulp capping consists of application a biocompatible material on the pulp which has been exposed unintentionally while removing caries or by traumatic injuries. The aim is to seal the pulp against bacterial leakage and provoking development of a dentinal bridge or barrier to obstruct the exposed area, and save the vital pulp underneath (1). Studies have shown that both bacteria and toxicity of materials could damage the pulp (2). Therefore, sealing ability and material toxicity are important factors in the prognosis of pulpal response to vital pulp treatment (2,3).

Historically, pulp capping was first performed in 1765 by Philip Pfaf using gold foil. In 1923, Davis suggested using the complex of Zinc Sulphate and Calcium Sulphate with Zinc Oxide for direct pulp capping. Calcium Hydroxide [Ca(OH)_2_] was used for pulp capping for the first time by Hermann (1930) and from the early 1940's till now it has been the most used material for pulp capping (1,4,5,6). Numerous studies have shown dentinal bridge formation in about 50 to 81 percent of cases treated by Ca(OH)_2 _complexes (7,8).

Also, it has been reported that when using pure Ca(OH)_2_ on the pulp, not only acts as a biologic barrier but also has some pulp tissue destruction. Other studies have shown that Calcium Hydroxide has a high toxic effect on cells in tissue culture. Inappropriate sealing of the exposed pulp is one of its greatest disadvantages (9,10,11). These harmful features have caused efforts in finding a material that can provoke reparative dentinal bridge formation with less undesirable effects.

In 1995 MTA was introduced by Torabinejad for sealing all the existing pathways between the root canal system and the outer surface of the tooth (12). This material became known as an appropriate material for pulp capping because of its various good properties such as high sealing effect and high PH, biocompati-bility, long-term stability, prevention of bacterial leakage, stimulation of cementum, bone and dentin formation (1,12,13,14). With the advent of technology, today lasers have different types of applications in dentistry which depends on their type, power, energy and effects on the oral and dental tissues. One of its applications is in endodontics which includes relieving and decreasing dentinal hyper- sensitivity, changing and modification of dentinal structure, pulp vitality evaluation, pulp capping, pulpotomy, root canal sterilization and endodontic surgery (15,16,17). Using laser for DPC has been suggested even in inflammatory pulps because of succession in pulp healing by sterilization and stimulation of secondary dentin formation (16,18).

Because there was not any article about using Laser and MTA together on DPC, the purpose of this experimental study was to evaluate the response of the exposed pulps of canine teeth in cats after direct pulp capping by three treatment approaches and to assess the effects of laser.

## MATERIALS AND METHODS

A total of 36 healthy mature permanent maxillary and mandibular canine teeth of 9 cats (Iranian mix, 3.5-5 kg weight) were chosen for this experimental study. Ten mg/kg Ketamine Hydrochloride (Alfasan, Holland) and 1.5 mg/kg Rampan (Xylazin, Alfasan, Holland) as a muscle relaxant were used for anesthesia by intramuscular injection. Then, the teeth health was examined and in the case of being intact, periapical radiographs were taken so that if they were healthy and their apices were mature, they would be chosen for the study. After local anesthesia by lidocain 2% (Darupakhsh, Tehran, Iran) the pulps were exposed at the buccal surface by a high speed handpiece and water spray using a diamond bur (801, W&Z, Germany) with a terminal diameter of 1 millimeter in isolated condition by using a rubber dam. Bleeding was controlled by normal saline rinse, and then the teeth were directly pulp capped by three methods:

A) First group: 12 teeth: capping the exposed area with 0.5-0.7 mm thickness of MTA (ProRoot, Dentsply, Tulsa, OK, USA).

B) Second group: 12 teeth: using Er:YAG laser (Fidelis, Fotana d.d, Ljubliana, Slovenia) featuring: wavelength=2.94nm, energy=200mJ, with a pulse duration of 700 µs which commercially is called very long pulse (VLP), frequency=3Hz, 15 seconds of direct projecting without air and water spray using a 0.6 mm fiber delivery system at the center and around the exposed area, then using 0.5-0.7 mm thickness of MTA as capping.

C) Third group: 12 teeth: using laser projection with the above features and then using 0.5-0.7 mm thickness of Ca(OH)2 (Dycal, Dentsply, Milford, USA) on the exposed area.

After base placement the remaining cavities were filled with Amalgam (2-4 mm) (Cinalux, Tehran, Iran).

Two months after the treatment, the animals were evaluated clinically and radiographically under anesthesia and their data were recorded. After 4 months the animals were sacrificed by vital perfusion for histological evaluation. Taking block sections of the teeth and the surrounding structures, the specimens were preserved in 10% formalin. Then the samples were transferred to a pathology laboratory. After decalcification, tissue processing, paraffin embedding, serial sectioning with 4-6 µm thickness, staining (H & E) and mounting the samples and coding them, a pathologist evaluated the sections using a high power field microscope with 400x magnification in a blind method.

**Figure1:**
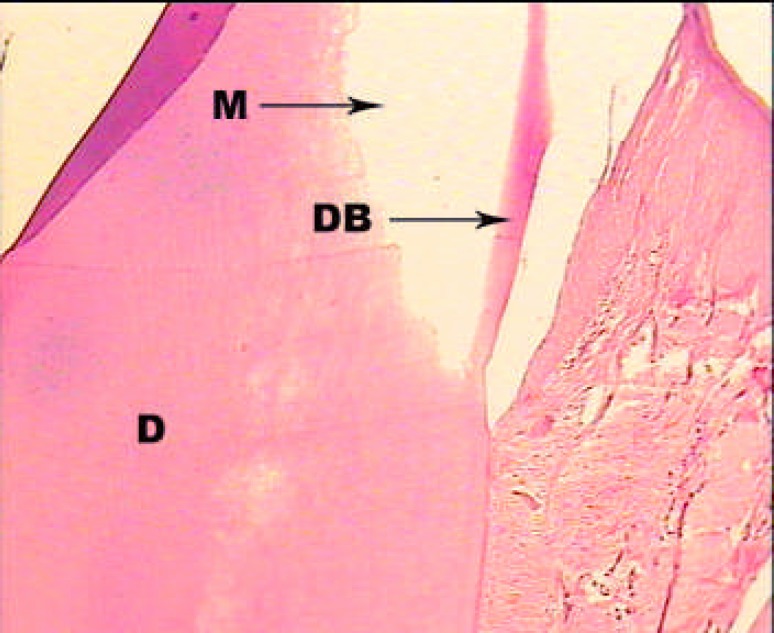
A sample treated with MTA alone; H and E staining: ×100. Dentinal bridge formation is seen near by the capping material (M: Material, DB: Dentinal Bridge, D: Dentin).

Histological evaluation was done in three fields of: 

A) Cellular inflammatory reaction including the type and intensity of the inflammation 

B) Pulpal soft tissue changes including hyperemia, necrosis and odontoblastic layer condition.

C) Pulpal hard tissue changes or dentinal bridge formation including location, continuity and thickness.

The evaluation was according to the scales defined as below:

**A-** Inflammation:

*a)* Type:

 0) without inflammation

 1) Acute inflammation

 2) Chronic inflammation

 3) Combination of acute and chronic inflammation.

*b)* Intensity:

 1) 0-30 inflammatory cells

 2) 30-60 inflammatory cells

 3) More than 60 inflammatory cells.

**B-** Soft tissue changes:

*a)* Hyperemia:

 1) 1-3 blood vessels

 2) 3-5 blood vessels

 3) More than 5 blood vessels.

*b)* Necrosis:

 0) without necrosis

 1) Signs of necrosis.

*c)* Odontoblastic layer cells:

 0) No odontoblastic layer cell.

 1) Presence of odontoblastic layer cells.

**C-** Dentinal bridge or hard tissue bridge:

*a)* Location: 

 1) Direct contact with the capping material.

 2) Close proximity with the capping material.

 3) No evidence of hard tissue formation.

*b)* Continuity:

 1) Complete 

 2) Slight contact of capping material with pulp. 

 3) Absence of dentinal bridge.

 c) Thickness: 

 1) More than 250 µm or 0.25 mm. 

 2) From 150 to 249 µm. 

 3) Less than 149 µm or absence of dentinal bridge. 

**Figure2:**
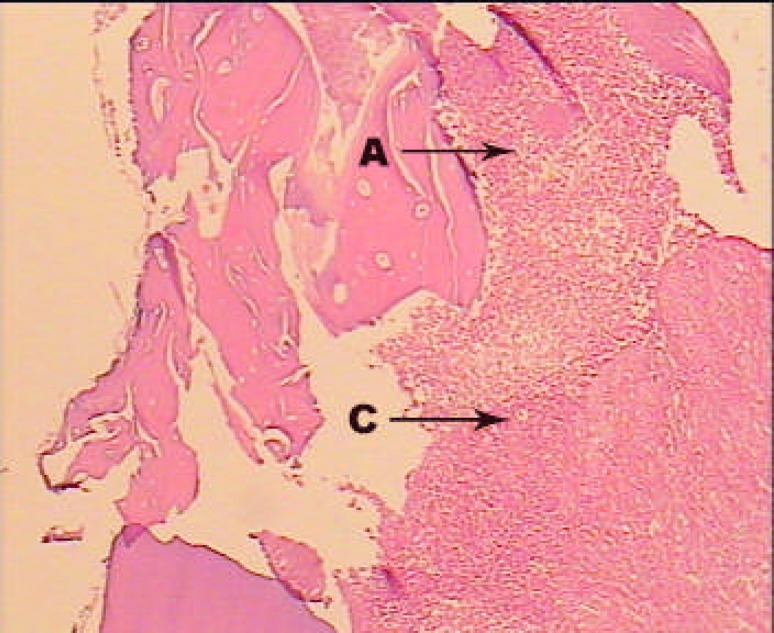
A sample treated with MTA alone; H and E staining; ×100. Acute and chronic inflammatory cells without any centre of necrosis (A: Acute inflammatory cells, C: Chronic inflammatory cells).

Histological evaluation of inflammatory reactions was performed on the most inflammatory sites.

Finally, the recorded data was tested using the SPSS software and significant level of 95%. Mann-Whitney test was used for comparing inflammation, dentinal bridge and hyperemia and the Chi-Square test for necrosis and odontoblastic cell layer.

## RESULTS

During the study, 4 teeth were excluded from study because of fracture. Table 1 and Table 2 show the number of specimens in each group, histologic responses and the mean and range of changes in the three different treatment modalities. The histological variables are mentioned in the materials and methods.

Comparison of histological evaluations showed no statistically significant difference between groups.

In first group, dentinal bridge was formed in more than 80% of the cases mostly specialized with the scale 2 (close proximity with the capping material). Also, the dentinal bridge thickness was mostly the scale 2 and 3 (less than 250 µm) (Figure 1). Inflammation under the capping material was mostly (36.4%) scale 2 (chronic) and scales 3 (combination of acute and chronic), respectively. Inflammation intensity in half of the cases (45.5%) was scale 1 (less than 30 inflammatory cells). In nearly 2/3 of the cases (63.6%) pulp necrosis was not reported (Figure 2).

**Figure3:**
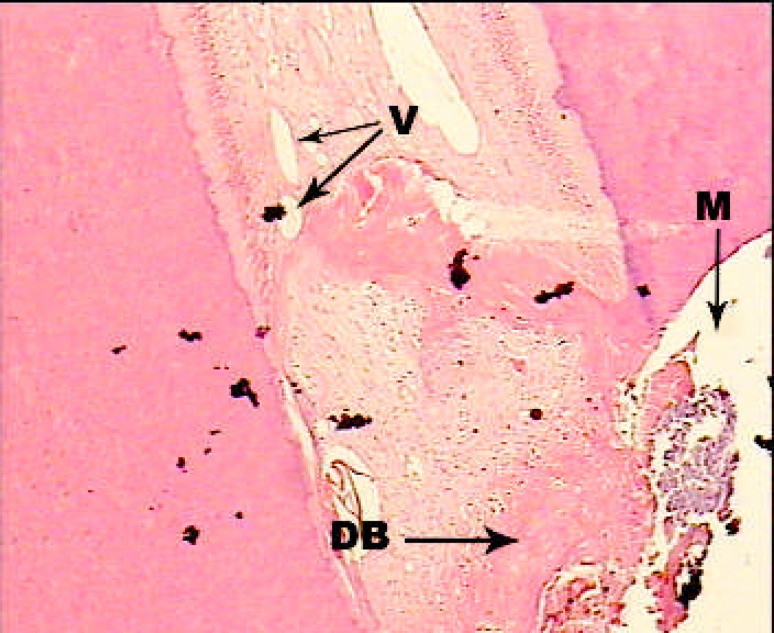
A sample treated with Laser + MTA; H and E staining; ×100. Dentinal bridge formation in complete form and thickness (V: Vessel, M: Material, DB: Dentinal Bridge).

**Figure4:**
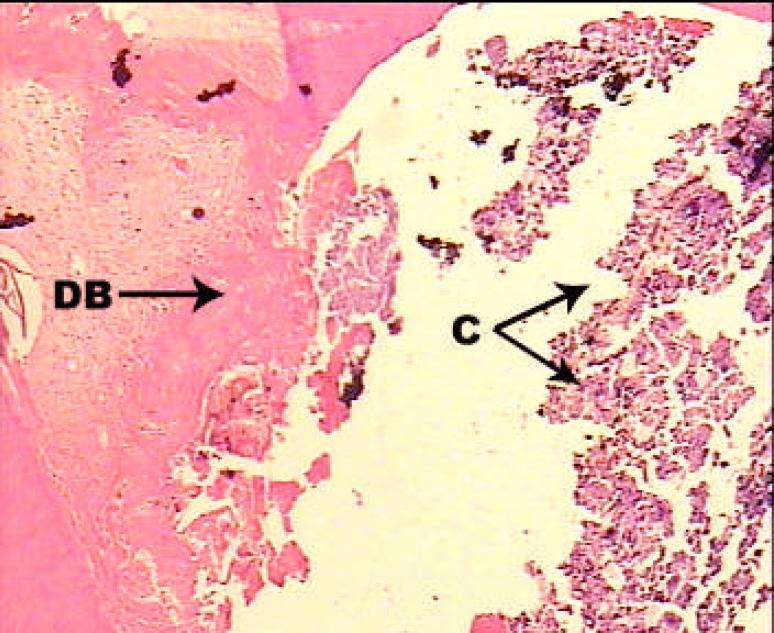
A sample treated with Laser + MTA; H and E staining; ×100. Chronic inflammatory cells underneath the dentine (DB: Dentinal Bridge, C: Chronic inflammatory cells).

In second group, dentinal bridge was developed in 80% of the cases that was mostly in close distance to the capping material. Interestingly in half of the cases continuity was scale 1 and in the rest of the cases it was scale 2 and 3. Thickness of the formed dentin was reported scale 1 (more than 250 µm) in more cases than were in first group (Figure 3). The chronic inflammation was in more cases (40%) than first group and the intensity of the inflammation was mostly scale 1 (less than 30 inflammatory cells). Also in 60% of the cases pulp necrosis was not observed (Figure 4).

In third group, dentinal bridge formation was observed in more than 60% of the cases.

**Figure5:**
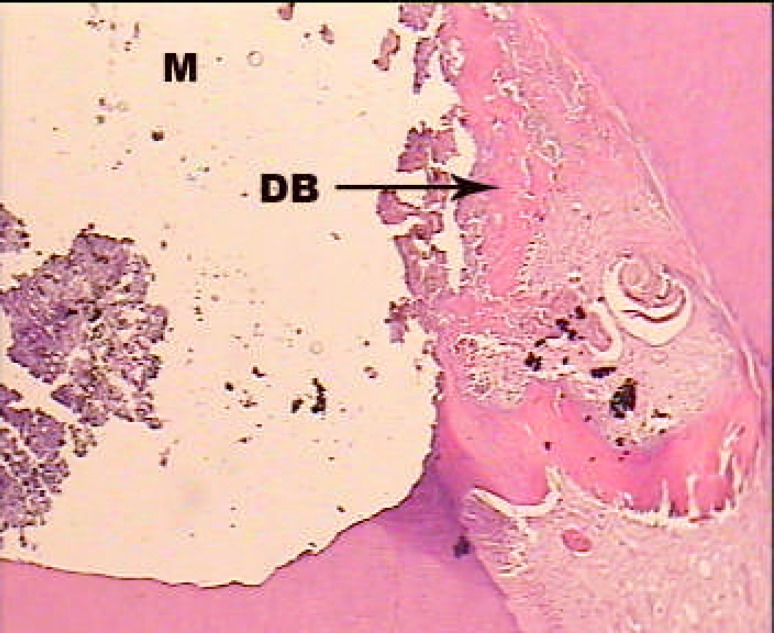
A sample treated with Laser + Ca(OH)2; H and E staining; ×100. Partially Dentinal bridge formation in less thickness (M: Material, DB: Dentinal Bridge).

**Figure 6:**
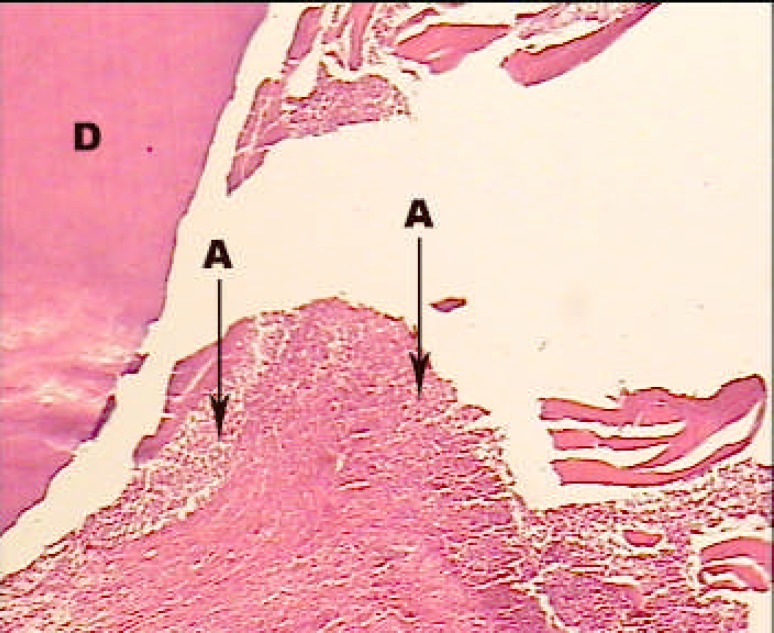
A sample treated with Laser + Ca(OH)2; H and E staining; ×100. Multinuclear inflammatory cells and inflammation with more density could be seen (A: Acute inflammatory cells, D: Dentin).

Surprisingly, dentin thickness and continuity compared to the former groups were more reported scale 3 (Figure 5). Also acute inflammation was reported underneath the dentinal bridge in 45.5% of cases. The intensity of inflammation was mostly scale 2 (45.5%). Another interesting fact was the presence of necrosis in more than half of the cases (54.4%) (Figure 6).

## DISCUSSION

The result of this pulp capping study showed that all experimental group developed barrier over capping area.

The inflammatory reactions including the type and intensity were evaluated in three treatment modalities. Although their differences were not statistically significant, but it could be mentioned that the two modalities: MTA alone and Laser+MTA showed slightly better inflammatory conditions comparing to Laser+Ca(OH)_2_. Also the Laser+MTA had the least inflammation intensity in more cases (50%) and consequently the MTA alone (45%). Some studies showed less inflammatory reaction after using MTA rather than using Ca(OH)_2_ (1,19- 22) that confirm the results of our study.

Some criteria such as hyperemia, necrosis and odontoblastic cellular layer were studied. These variables demonstrate the vitality status of the pulp. The results of this study showed that using MTA alone and Laser+MTA had a better biocompatibility than Laser+Ca(OH)_2_, although this difference wasn't statistically significant. Aeinechi *et al* showed that hyperemia and necrosis in the MTA group was much less than the Ca(OH)_2_ group and the odontoblastic layer formation was higher.(19) Moritz *et al* stated that pulp vitality and blood flow was preserved in 93% of cases using Laser+Ca(OH)_2_ and in 66.6% of cases using Ca(OH)_2_ alone (23). This finding was confirmed by Satucci *et al* (24).

**Table 1:** Distribution of relative frequency in different groups according to histologic criteria

**Treatment method**				**(A)** **#11**	**(B)** **#10**		**(C)** **#11**	
**Histologic evaluation**			**S**	**Relative frequency (%)**				
Dental pulp	Inflammation	Type	0	27.3		20		9.1
			1	9.1		10		45.5
			2	36.4		40		9.1
			3	27.3		30		36.4
		Intensity	1	45.5		50		36.4
			2	36.4		30		45.5
			3	18.2		20		18.2
	Hyperemia		1	36.4		40		36.4
			2	45.5		40		36.4
			3	18.2		20		27.3
	Necrosis		0	63.6		60		45.5
			1	36.4		40		54.5
	Odontoblastic layer		0	54.5		60		63.6
			1	45.5		40		36.4
Dentinal barrier	Condition		1	18.2		20		18.2
			2	63.6		60		45.5
			3	18.2		20		36.4
	Continuity		1	18.2		30		9.1
			2	54.5		50		45.5
			3	27.3		20		45.5
	Thickness		1	27.3		40		0
			2	36.4		30		63
			3	36.4		30		36.4

According to this study and other similar studies, it seems that using MTA alone or with Laser has better results in saving pulp vitality and preventing necrosis, comparing to using Ca(OH)_2_ with Laser.

The location, continuity and thickness of the dentinal bridge were evaluated. The formation of dentinal bridge, basically, could be considered as a sign of successful pulp healing.

According to this study, it looks that using Laser+MTA is slightly better in location, continuity and thickness than using MTA alone or Laser+Ca(OH)_2_ (Table 1 and Table 2). But, this difference was not statistically significant. Many researchers emphasized on the ability of MTA in dentinal bridge formation (1,25,26). Also Aeinechi *et al* emphasized on the advantage of MTA in dentinal bridge formation comparing to Ca(OH)_2_ (19). This positive feature of MTA can be related to its good sealing ability, biocompatibility and less toxicity (10,21). On the other hand, Melcer showed that Co_2_ Laser can be effective for dentinal bridge formation in pulp capped teeth because of its good thermal effects and sterilization features (27). Jayawardena *et al*. showed that pulpal tissues of teeth that were exposed with Er:YAG Laser have the ability of dentinal bridge and reparative dentin formation (28).

## CONCLUSION

According to this study, it looks like that using laser doesn't have much effects in direct pulp capping treatment and the type of capping material (Ca(OH)_2_ or MTA) doesn’t make any differences. Other studies with more specimens may be required to find the differences more accurately.

**Table 2:** Mean, median and range of histologic criteria in different groups.

**Method**			**MTA(A) n=11**			**Las+MTA(B) n=10**			**Las+Ca(OH)**_2_ **(C) n=11**		
**Criteria**			**Mean**	**Median**	**Range**	**Mean**	**Median**	**Range**	**Mean**	**Median**	**Range**
Dental pulp	Inf	Type	1.63	2	3	1.80	2	3	1.73	1	3
		Intensity	1.73	2	2	1.70	1.5	2	1.82	2	2
	Hyperemia		1.82	2	2	1.80	2	2	1.91	2	2
	Necrosis		0.36	0	1	0.40	0	1	0.55	1	1
	Odont. layer		0.46	0	1	0.40	0	1	0.36	0	1
Dentinal barrier	Condition		2.00	2	2	2.00	2	2	2.18	2	2
	Continuity		2.09	1	1	1.90	2	2	2.36	2	2
	Thickness		2.09	2	2	1.90	2	2	2.36	2	1

It could be stated that because of performing the study under sterile condition and controlled variables, the results of laser, that are mostly related to the sterilization and disinfection effect were not apparent. It is recommended that other studies be done closer to clinical situations (like trauma-induced exposures).
